# Supplementary figures and images for: Paternal-effect-genes revealed through sperm cryopreservation in Perca fluviatilis
Source: Sci Rep. 2024 Mar 16;14:6396. doi: 10.1038/s41598-024-56971-w (PMC10944473; doi:10.1038/s41598-024-56971-w)

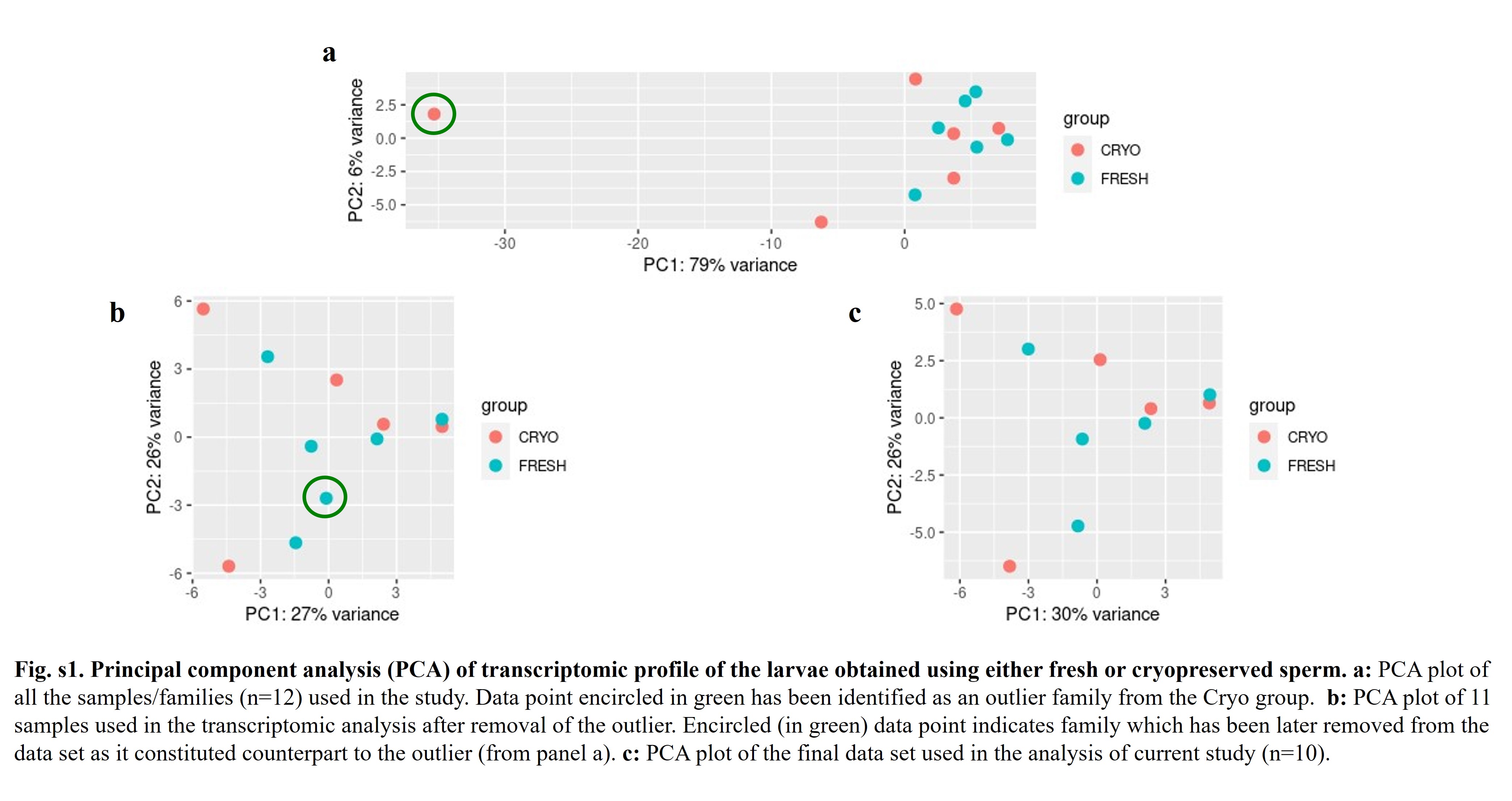

Supplement: Supplementary file 1 — Supplementary Figure S1. [file 41598_2024_56971_MOESM1_ESM.jpg]

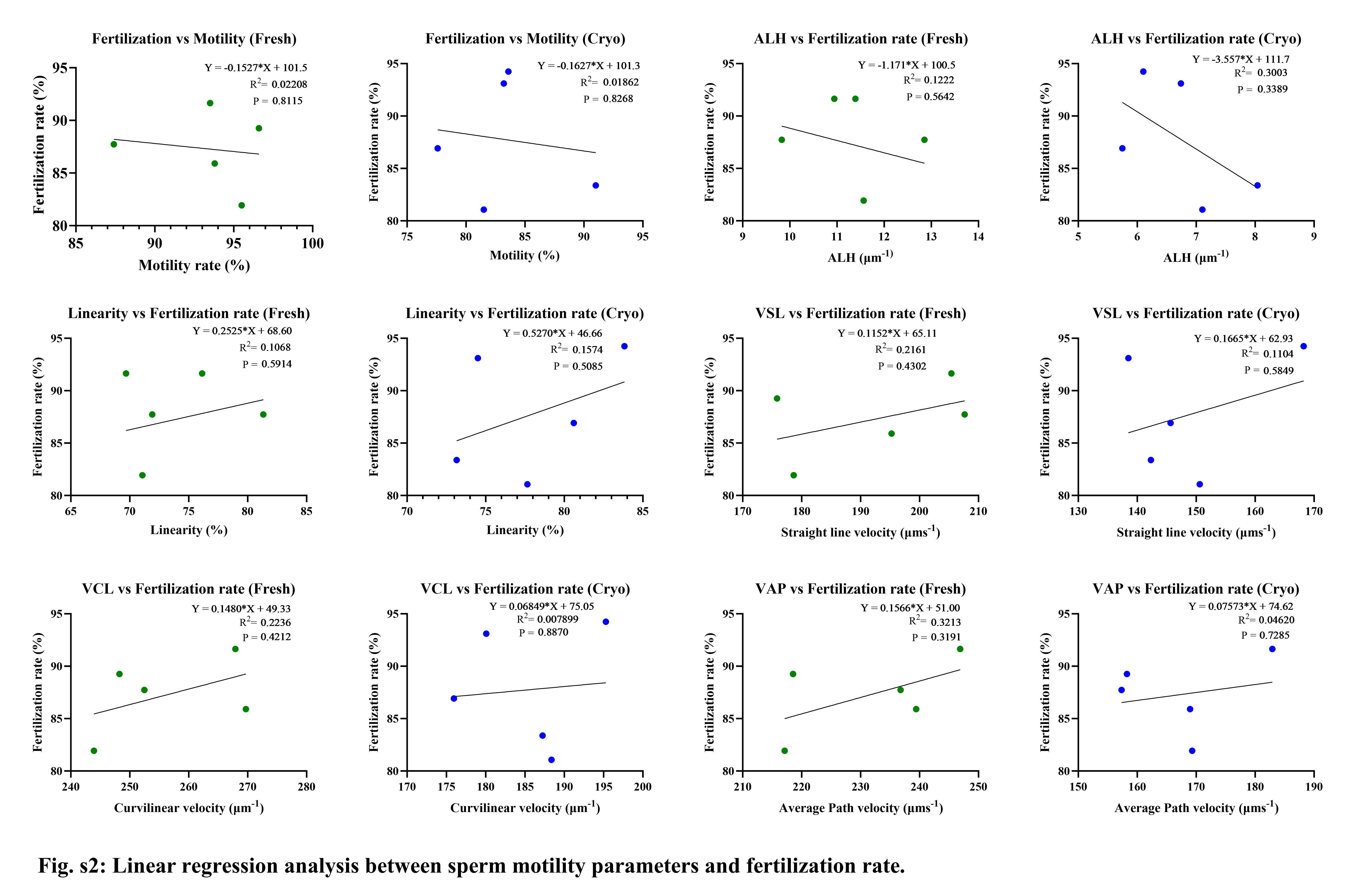

Supplement: Supplementary file 2 — Supplementary Figure S2. [file 41598_2024_56971_MOESM2_ESM.jpg]
