# Supplementary material for: Paternal-effect-genes revealed through sperm cryopreservation in Perca fluviatilis
Source: Sci Rep. 2024 Mar 16;14:6396. doi: 10.1038/s41598-024-56971-w (PMC10944473; doi:10.1038/s41598-024-56971-w)
Supplement: Supplementary file 3 — Supplementary Figure S3. [file 41598_2024_56971_MOESM3_ESM.pdf]

Fig. s3: Expression values, measured in TPMs and expressed as log fold changes, of differentially expressed genes (DEGs) identified in larvae of the Eurasian perch, obtained using either cryopreserved or fresh sperm, across various tissues in evolutionarily distinct fish species.

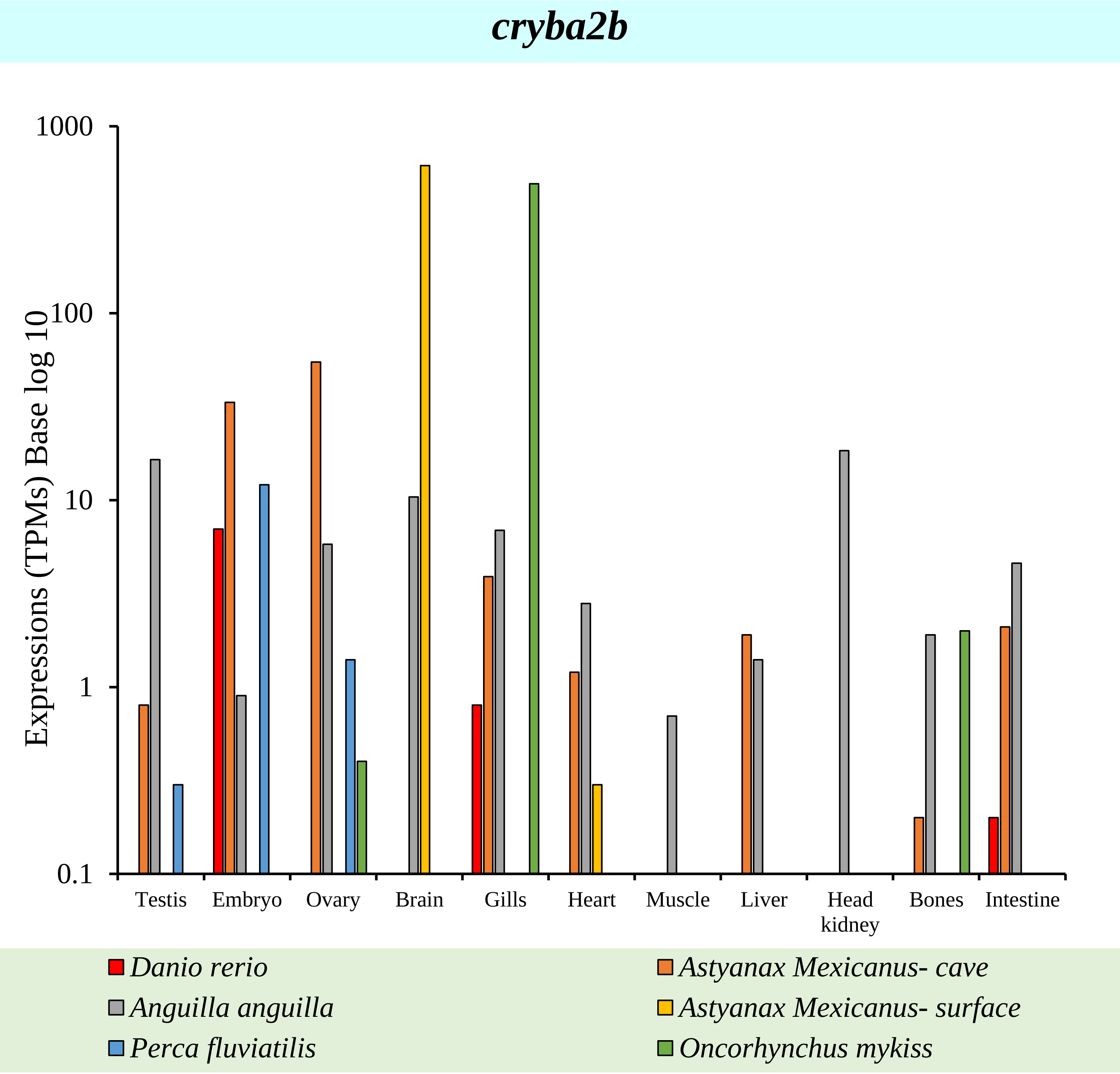

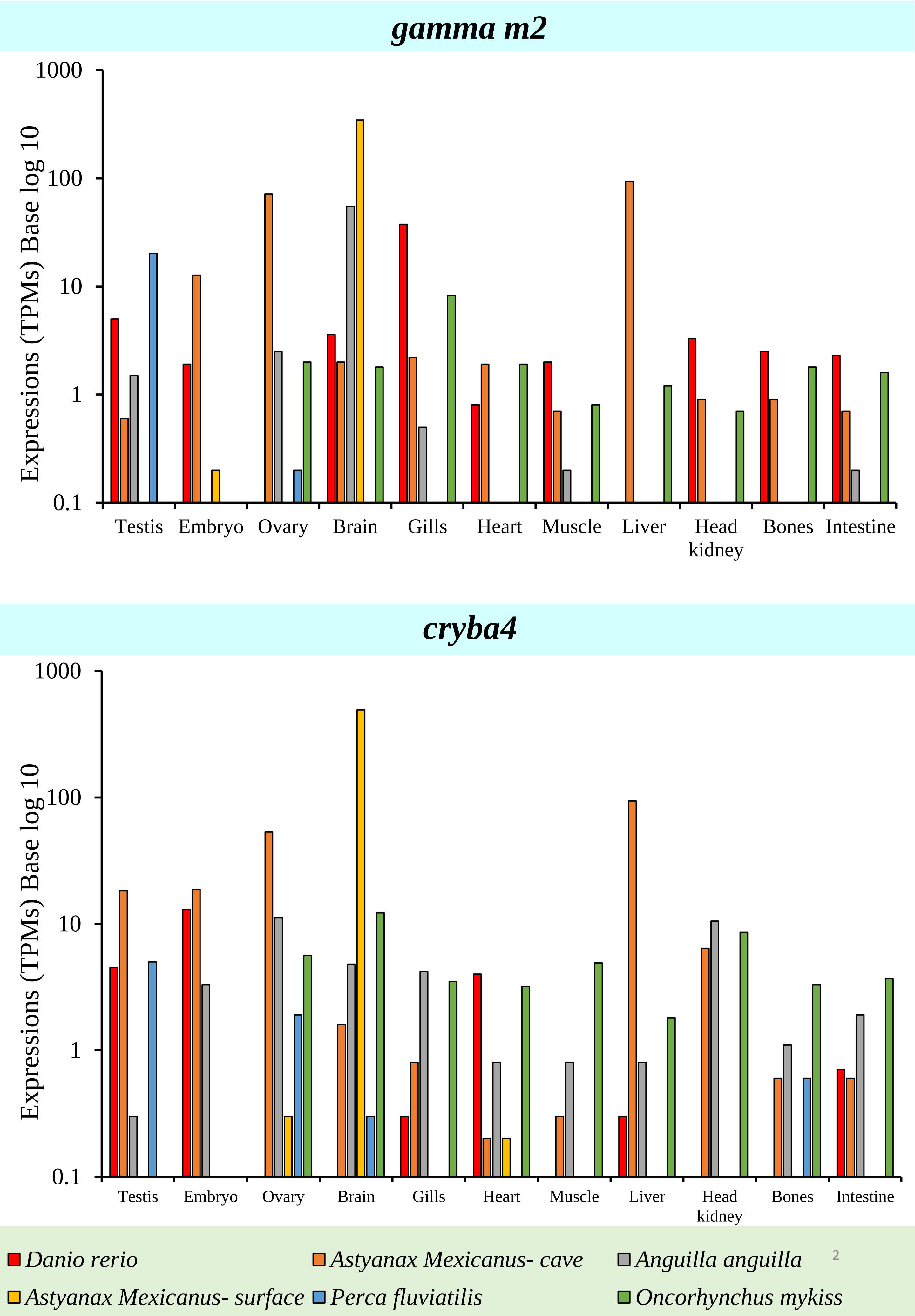
gamma m2
Expressions (TPMs) Base log 10
1000
100
10
1
0.1
Testis
Embryo
Ovary
Brain
Gills
Heart
Muscle
Liver
Head kidney
Bones
Intestine
cryba4
Expressions (TPMs) Base log 10
1000
100
10
1
0.1
Testis
Embryo
Ovary
Brain
Gills
Heart
Muscle
Liver
Head kidney
Bones
Intestine
Danio rerio
Astyanax Mexicanus- cave
Anguilla anguilla
Astyanax Mexicanus- surface
Perca fluviatilis
Oncorhynchus mykiss

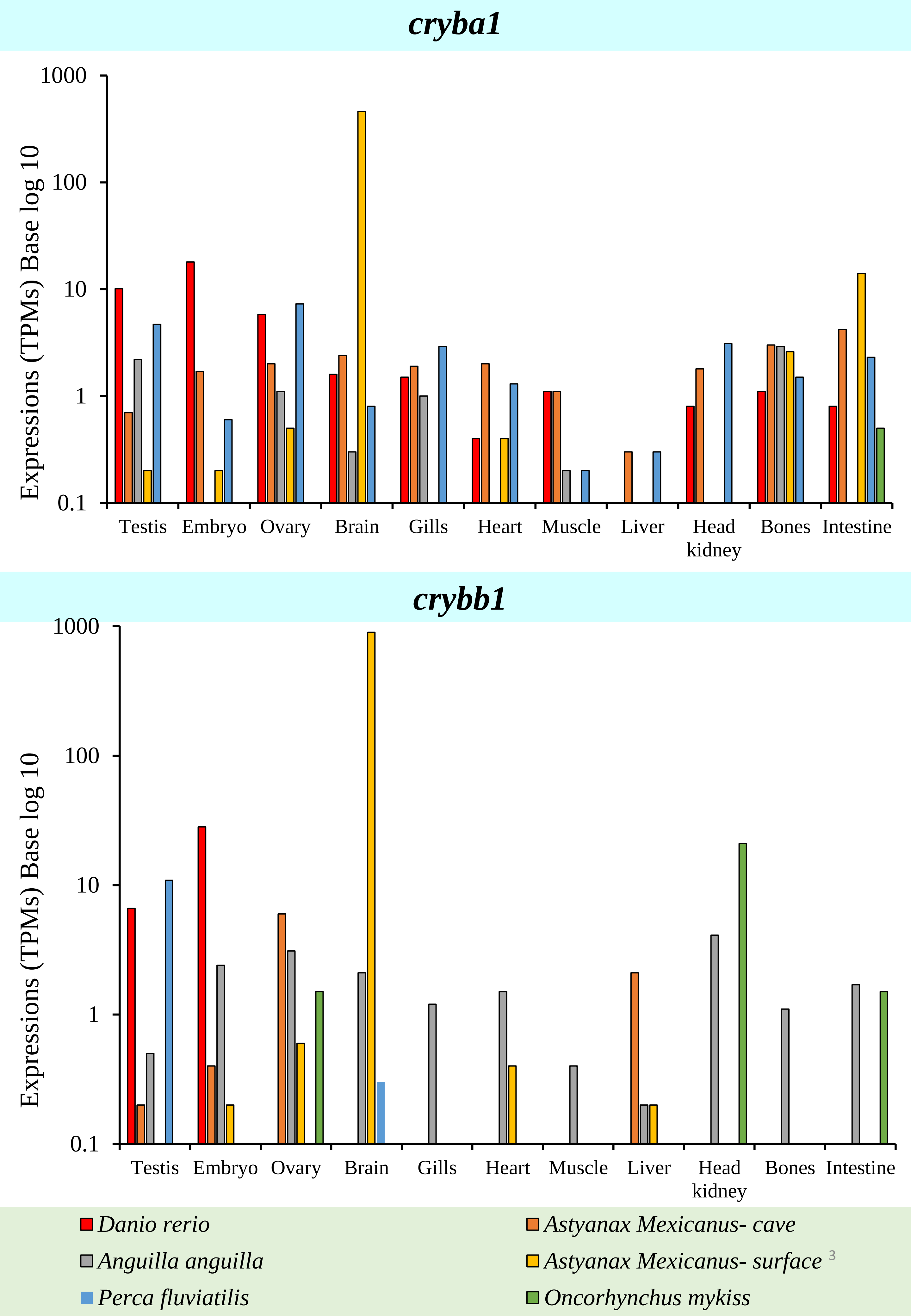

cryba1
Expressions (TPMs) Base log 10
1000
100
10
1
0.1
Testis
Embryo
Ovary
Brain
Gills
Heart
Muscle
Liver
Head kidney
Bones
Intestine
crybb1
Expressions (TPMs) Base log 10
1000
100
10
1
0.1
Testis
Embryo
Ovary
Brain
Gills
Heart
Muscle
Liver
Head kidney
Bones
Intestine
Danio rerio
Astyanax Mexicanus- cave
Anguilla anguilla
Astyanax Mexicanus- surface
Perca fluviatilis
Oncorhynchus mykiss

rbp4l

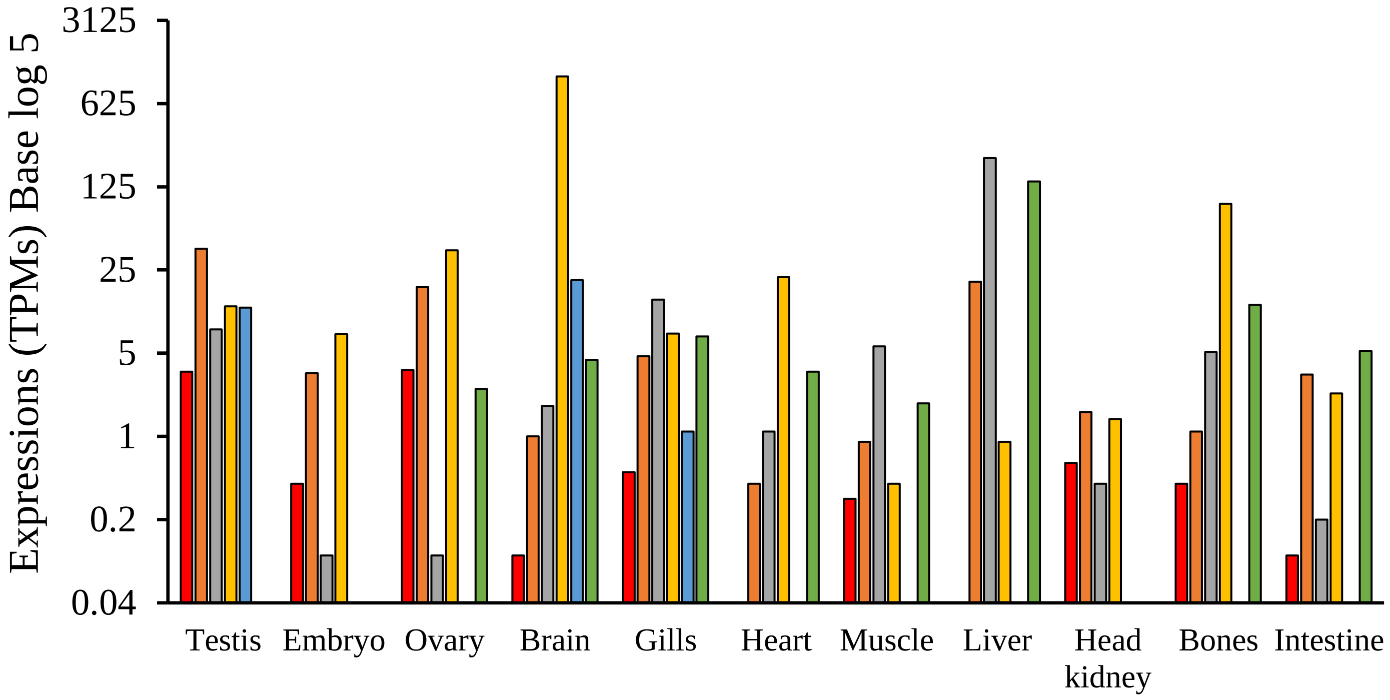

crygmxl2

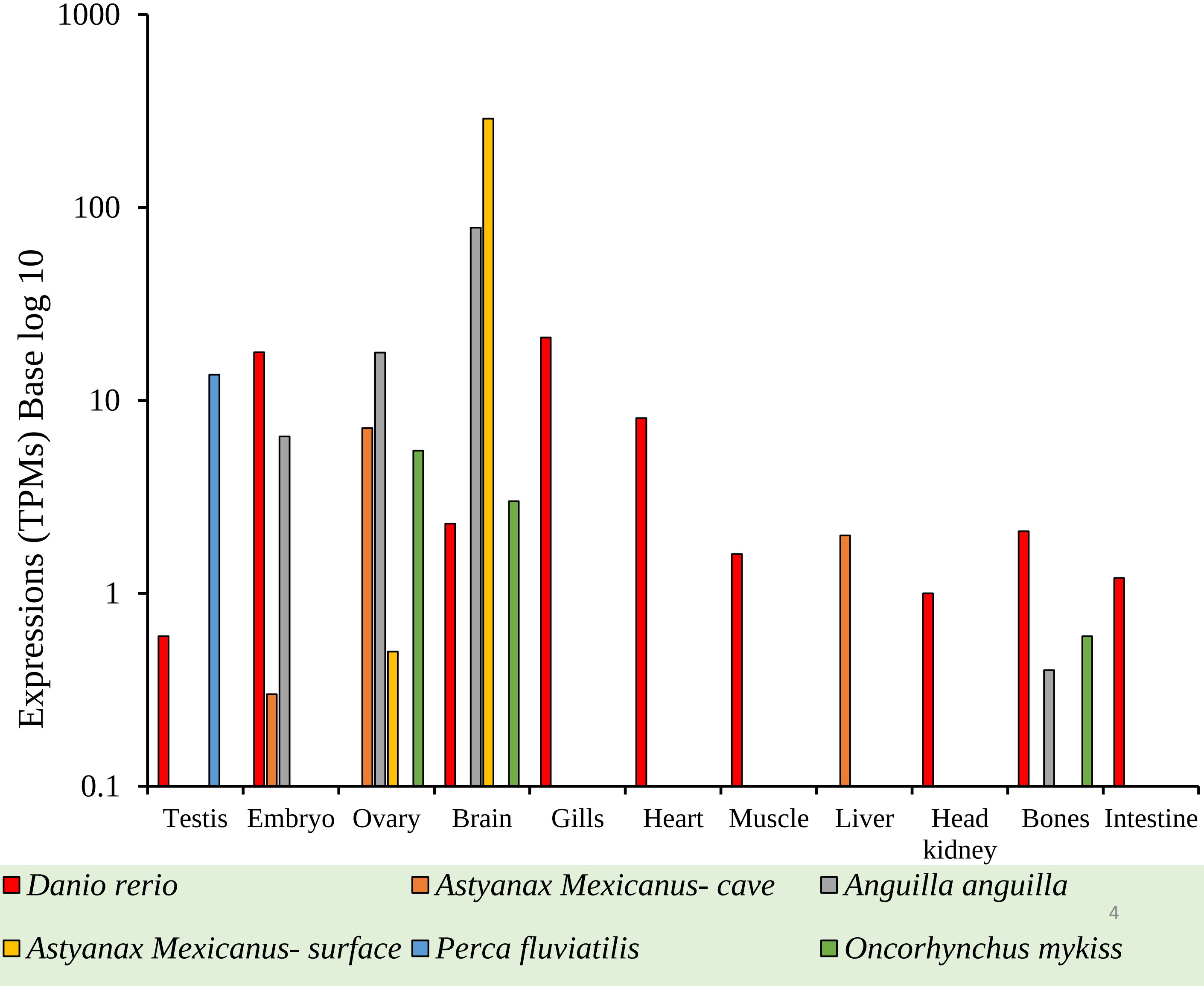

tgfbi

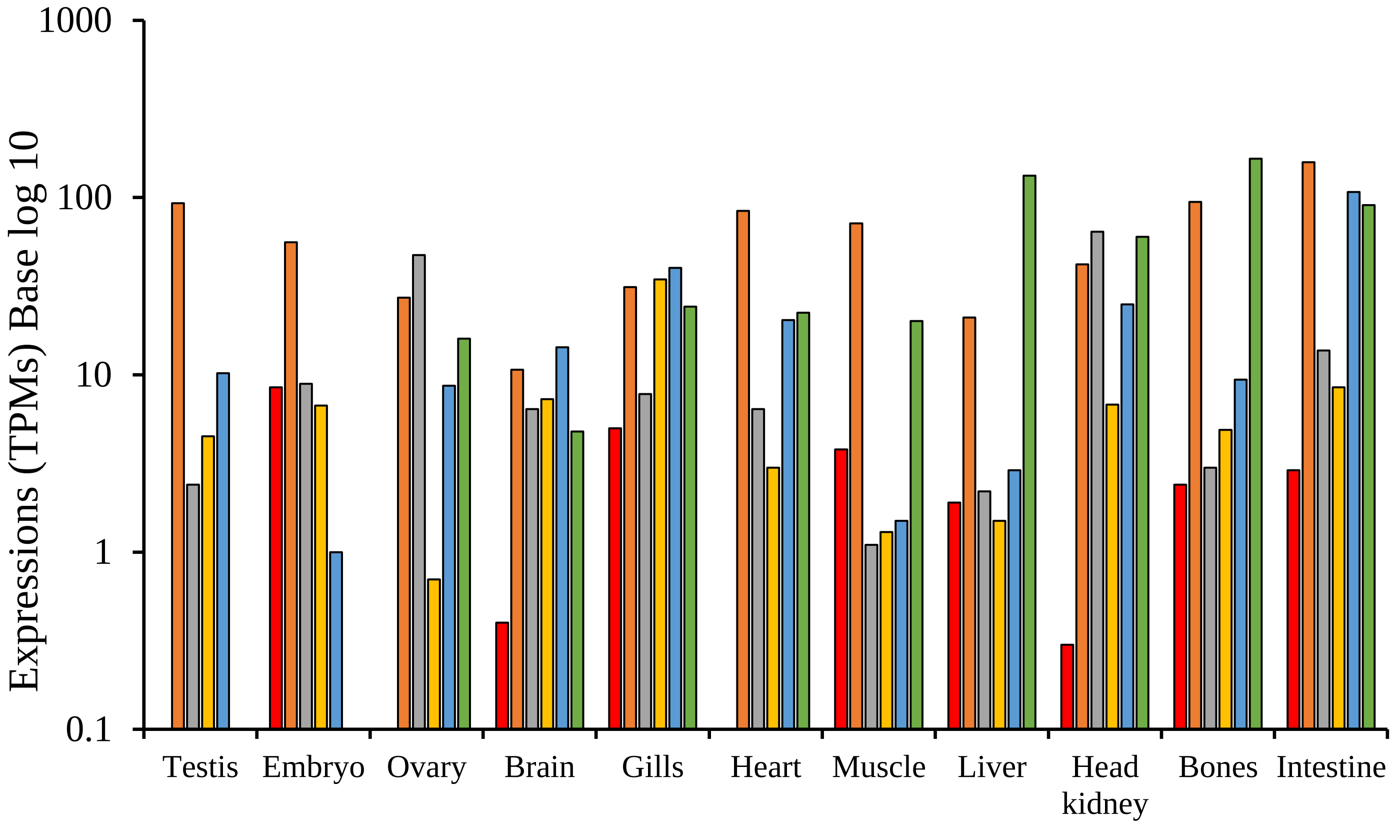

crygm3

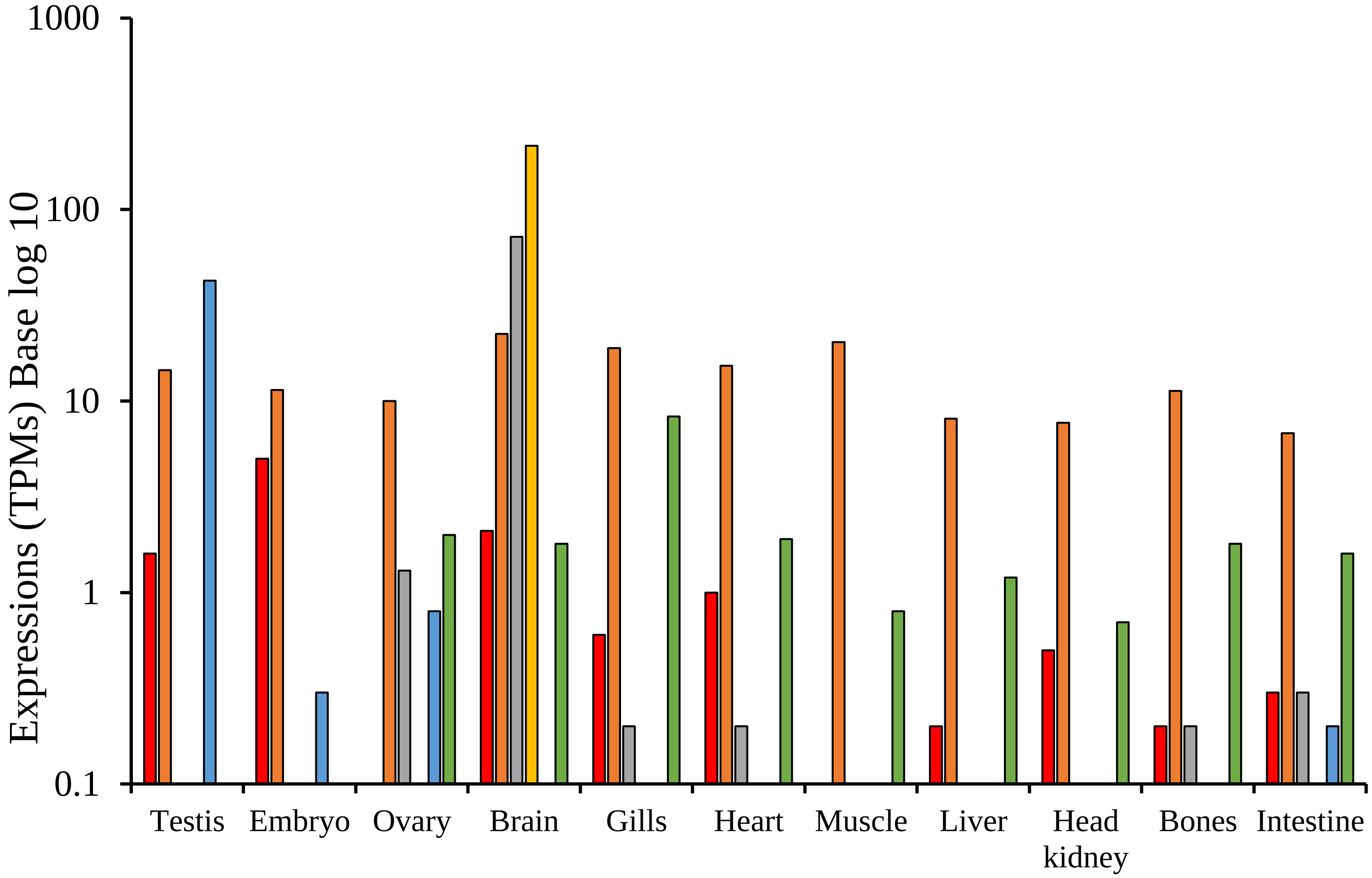

Danio rerio | *Astyanax Mexicanus- cave* | *Anguilla anguilla*

Astyanax Mexicanus- surface | *Perca fluviatilis* | *Oncorhynchus mykiss*

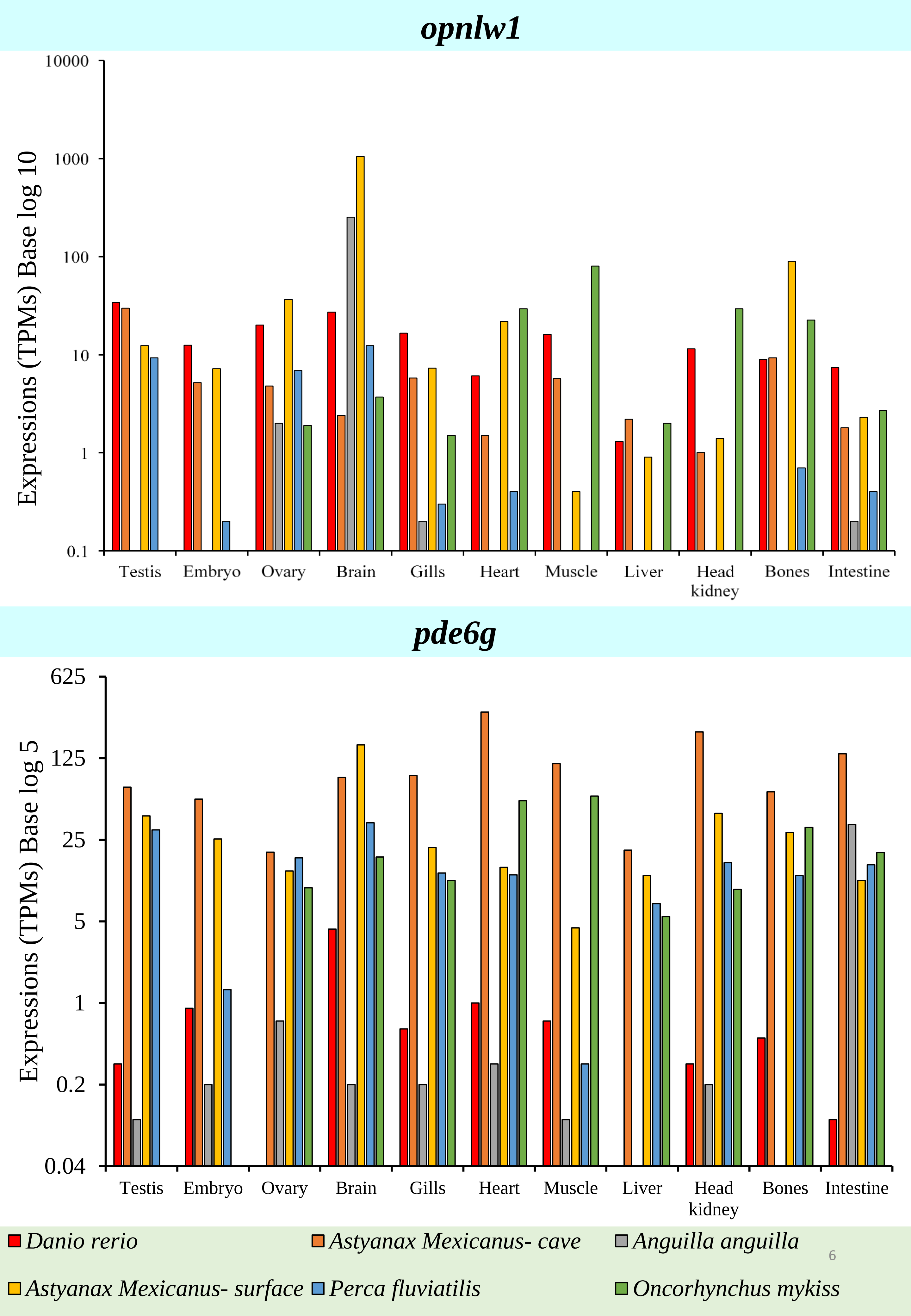

opnlw1
Expressions (TPMs) Base log 10
10000
1000
100
10
1
0.1
Testis
Embryo
Ovary
Brain
Gills
Heart
Muscle
Liver
Head kidney
Bones
Intestine
pde6g
Expressions (TPMs) Base log 5
625
125
25
5
1
0.2
0.04
Testis
Embryo
Ovary
Brain
Gills
Heart
Muscle
Liver
Head kidney
Bones
Intestine
Danio rerio
Astyanax Mexicanus- cave
Anguilla anguilla
Astyanax Mexicanus- surface
Perca fluviatilis
Oncorhynchus mykiss
