# Supplementary material for: Paternal-effect-genes revealed through sperm cryopreservation in Perca fluviatilis
Source: Sci Rep. 2024 Mar 16;14:6396. doi: 10.1038/s41598-024-56971-w (PMC10944473; doi:10.1038/s41598-024-56971-w)
Supplement: Supplementary file 5 — Supplementary Table S2. [file 41598_2024_56971_MOESM5_ESM.pdf]

Table s2: Details of the primers used for qRT PCR in the study.

Gene name	Sequences	Amplicon length (bp)
crystallin, beta A2b [cryba2b]	F: ACAAGATCCGCTCCATCAAG	167
	R: GATGGGTCTGAAGGAAAGCA	
crystallin beta A4 [cryba4]	F: GCTATGAGCACGCCTCCTAC	173
	R: CTCACGCTCGTAGATGGTCA	
crystallin beta B1 [crybb1]	F: CATGATGTTCGACCAGGAGA	153
	R: TCCCCACGGAAGTTAGTCTG	
crystallin, gamma MX, like 2 [crygmxl2]	F: TAACTGCTGCAACTCCATGC	134
	R: AGTTGTTGAAGCCCATCCAG	
retinal cone rhodopsin-sensitive cGMP 3',5'-cyclic phosphodiesterase subunit gamma-like [pde6g]	F: AGACCGGACACAAACTGACC	140
	R: GGTCCTCTGCTTGAACTTGG	
red-sensitive opsin-like [opn1lw1]	F: CCAGGCGGTACAATGAAGAT	105
	R: GCGGAGCAATGTGGTAATTT	
gamma-crystallin M2-like [gamma M2]	F: GGGCAACCAGTACTTCCTGA	188
	R: CCATGACGTTGTCACAGTCC	
beta-crystallin A1-like [cryba1]	F: TACAGCGGTTCCCTCTCCTA	218
	R: AGCCAACTTCAGGCATCATC	
gamma-crystallin M3-like [crygm3]	F: GGAGAACTTCGGTGGTCAGA	138
	R: CCTCTGTAGTTGGGCTGCTC	
retinol binding protein 4, like [rbp4l]	F: TTTGACCCCAAGAGGTATGC	165
	R: ACACAACCCAGAAGCCAAAC	
transforming growth factor beta induced [tgfbi]	F: CTGAAGGAGCGTCTGTCCTC	146

	R: AAACGTCCGGTCTTATCGTG	
tetraspanin 7 [tspan7]	F: CACCAACTGCTCACCAGAGA	179
	R: ACAAGCAGCAGGACAGGAAT	
cytochrome c-like, transcript variant X1 [cycs]	F: TGTGGAGAATGGAGGAAAGC	124
	R: ATTCCAGACAATGCCTTTGC	
ER membrane protein complex subunit 10, transcript variant X2 [emc10]	F: GCCCAGCGTCTCACTAACTC	172
	R: GGCTCTGACAAATGCTGTGA	
pre-mRNA-splicing factor [syf2]	F: GGAAGTTGTGGAGGAGGACA	167
	R: CTGCATCGTCAGCAGTGATT	
ER membrane protein complex subunit 3-like [emc3]	F: AACTGGGCCTTCTCTGGATT	150
	R: CCCAAACACGTTGAGGAAGT	
